# Development of a Natural Coating Based on Fermented Milk Whey for Biopreservation of Cheese

**DOI:** 10.3390/foods14132149

**Published:** 2025-06-20

**Authors:** Ana Moreno, Jorge Calpe, Victor Dopazo, Carlos Luz, Juan Manuel Quiles, Giuseppe Meca

**Affiliations:** Laboratory of Food Chemistry and Toxicology, Faculty of Pharmacy, University of Valencia, Av. Vicent Andrés Estellés s/n, 46100 Burjassot, Spain; ana.moreno@uv.es (A.M.); jorge.calpe@uv.es (J.C.); carlos.luz@uv.es (C.L.); giuseppe.meca@uv.es (G.M.)

**Keywords:** edible-coating, biopreservation, cheese, lactic acid bacteria, *Penicillium*, LAB-fermented whey

## Abstract

Consumer demand for natural, additive-free foods is increasing. Following the trend, this study evaluated the antifungal potential of lactic acid bacteria (LAB) against *Penicillium* species commonly found in cheese, using both LAB ferments and hydroxypropylmethylcellulose (HPMC) coatings. LAB strains were first screened with a dual-culture assay. Fermentations in Man–Rogosa–Sharpe (MRS) broth and milk whey were lyophilized and tested, with whey-based ferments showing greater antifungal activity. All whey ferments inhibited fungal growth, with KK_13_, KB_2_, KB_3_, and KB_4_ being the most effective based on MIC and MFC assays. KB_3_-fermented whey had the highest levels of antifungal metabolites, such as phenyllactic acid. A coating containing 5% HPMC and 100 g/L of KB_3_-fermented whey was applied to cheese slices, reducing the fungal counts of *Penicillium commune* by more than 1 Log_10_ CFU per gram and extending shelf life by 12 days. In whole-cheese trials with natural contamination, this coating delayed visible fungal growth until day 60, extending shelf life by 45 days compared with uncoated samples and 33 days compared with coated controls. These findings support the use of LAB-fermented whey and HPMC coatings as natural preservation strategies, thereby contributing to the sustainable reuse of dairy by-products.

## 1. Introduction

Food preservation aims to maintain food quality at a desired level, maximizing the nutritional benefits of the product while preventing spoilage. The primary objectives of food preservation include extending shelf life without compromising nutritional, organoleptic, or technological properties and preventing food deterioration [1]. Various techniques are employed to minimize chemical and microbial degradation of food, including traditional methods (e.g., drying, chilling, freezing, salting, smoking, and fermentation); classical techniques (e.g., sterilization, pasteurization, refrigeration, freezing, dehydration, lyophilization, and the use of additives); and modern techniques (e.g., microwaves, electrical pulses, aseptic packaging, high-pressure processing, and irradiation). Among these, classical preservation methods remain the most widely used as they effectively eliminate undesirable microorganisms. However, these methods may negatively impact the organoleptic and nutritional quality of food products [2].

Currently, the direct addition of food additives is one of the simplest preservation techniques used to extend shelf life, prevent microbial spoilage, preserve nutritional value, and improve the physical and chemical properties of food products [3]. Although food additives are deemed safe at permitted concentrations, consumer perception towards them remains negative, as they are often regarded as unnatural, unhealthy, or even a public health risk. This perception conflicts with the demand for “traditional” and “clean-label” products [4]. Additionally, some studies have highlighted the potential toxicity of certain food additives currently used in the industry, emphasizing the need for their periodic reevaluation [5].

The increasing consumer demand for natural food products and extended shelf life has prompted the search for alternatives to synthetic preservatives. One such alternative is biopreservation, which involves the use of naturally derived antimicrobial compounds from bacteria, fungi, or plants to inhibit foodborne pathogens and contaminant microorganisms [6]. In recent years, biopreservation using lactic acid bacteria (LAB) has gained significant interest [3]. LAB are classified as generally recognized as safe (GRAS) by the U.S. Food and Drug Administration (FDA) and as qualified presumption of safety (QPS) by the European Food Safety Authority (EFSA) [7,8].

LAB are a heterogeneous group of bacterial species (Gram-positive, non-sporulated, immobile, catalase negative, and tolerant anaerobic fermentatives) grouped from more than six families, among which the *Aerococcaceae*, *Carnobacteriaceae*, *Lactobacillaceae*, *Leuconostocaceae* and *Streptococcaceae* are the more recognized, with more than 40 genera, of which 7 are commonly associated with food: *Lactococcus*, *Enterococcus*, *Streptococcus*, *Leuconostoc*, *Weissella*, *Carnococcus*, and *Oenococcus* [9,10]. During fermentation, LAB produce organic acids that lower the pH, thereby inhibiting the growth of undesirable microorganisms. Additionally, LAB synthesize a wide range of bioactive compounds, including bacteriocins and antifungal metabolites, which help extend food shelf life while reducing the need for physicochemical treatments and synthetic additives. These properties ensure food safety without altering taste, aroma, texture, or functional characteristics [11]. The potential use of LAB and their antifungal metabolites isolated from whole-wheat sourdough dairy products, fermented dates, chicha and tocosh has been recently demonstrated [12,13,14,15].

In cheese production, LAB serve as the primary microbiota responsible for milk coagulation, imparting distinct organoleptic characteristics while contributing to shelf-life extension through pH reduction and competitive exclusion of spoilage microorganisms [16]. However, the secondary microbiota of cheese, which includes yeasts, fungi, and bacteria, can also harbor spoilage organisms. Filamentous fungi that proliferate during cheese storage generate undesirable flavors and reduce overall product quality [17]. The main fungal species contaminating cheeses belong to the *Penicillium* genus and include *P. commune*, *P. palitans*, *P. nalgiovense* and *P. verrucosum* [18].

Extending the shelf life of dairy products has long been a major concern for the food industry. Cheese is the most widely consumed solid dairy product in Europe and North America, with per capita consumption expected to increase over the next decade [19]. At the same time, whey, a by-product of cheese production, has historically been considered an industrial waste [20]. However, whey is rich in lactose (45–50 g/L), proteins (6–8 g/L), lipids (4–5 g/L), mineral salts, B vitamins, and organic acids, making it a valuable resource for the production of functional, nutritional, and technologically relevant compounds [21]. These include concentrated whey proteins and oligosaccharides with prebiotic properties, such as Galβ6GalNAcα3Galβ4Glc and Galβ6Galβ6Galβ6Glc [22].

There is increasing interest in the application of antimicrobial substances produced by LAB (e.g., lactic acid and phenyllactic acid), either directly or through delivery systems such as edible coatings and films, to inhibit the growth of spoilage and pathogenic microorganisms in food matrices [23]. Additionally, the efficient management and reuse of industrial by-products align with the United Nations’ Agenda 2030 for Sustainable Development, particularly Goal 12: “Responsible Consumption and Production” [24].

The present study aimed to evaluate the antifungal activity of whey fermented by various LAB strains against common cheese-contaminating fungi from the *Penicillium* genus and to assess its application in hydroxypropylmethylcellulose (HPMC) coatings for cheese biopreservation.

## 2. Materials and Methods

### 2.1. Chemicals and Reagents

Sweet goat whey and cheese was supplied by ALCLIPOR, S. A. L. (Benassal, Spain). Potato Dextrose Broth (PDB), Potato Dextrose Agar (PDA), MRS broth, and MRS culture media were purchased from Liofilchem Bacteriology Products (Roseto degli Abruzzi, Italy). Deionized water (<18 MΩ cm resistivity) used to make the culture media was obtained by a Milli-Q purification system (Millipore, Bedford, MA, USA). Mikrobiologie Anaerocult A strips were obtained from Merck KGaA (Darmstadt, Germany). HPMC was purchased from Sigma-Aldrich (St. Louis, MO, USA). Glycerol, acetonitrile, ethyl acetate, formic acid and methanol were from VWR Chemicals (Radnor, PA, USA). C_18_, magnesium sulfate (MgSO_4_), sodium chloride (NaCl), DL-3-phenyllactic acid, 3-4-dihydroxyhydrocinnamic, benzoic acid, vanillic acid, 1-2-Dihydroxybenzene, 3-(4-hydroxy-3-methoxyphenyl) propionic were acquired from Sigma-Aldrich (Dublin, Ireland).

### 2.2. Microbiological Culture

The *Penicillium* camemberti strains CECT 2267 (*P. camemberti* CECT 2267), *Penicillium* expansum CECT 2278 (*P. expansum* CECT 2278), *Penicillium roqueforti* CECT 2905 (*P. roqueforti* CECT 2905), *Penicillium digitatum* CECT 2954 (*P. digitatum* CECT 2954), *Penicillium brevicopactum* CECT 2316 (*P. brevicopactum* CECT 2316), *Penicillium nordicum* CECT 2320 (*P. nordicum* CECT 2320), *P. commune* CECT 20767, *Penicillium solitum* CECT 20818 (*P. solitum* CECT 20818) were obtained from the Spanish Type Culture Collection (CECT Valencia, Spain). The *Penicillium verrucosum* strain VTT D-01847 (*P. verrucosum* VTT D-01847) was obtained from the collection of the VTT Technical Research Center (Espoo, Finland). These cultures were maintained in sterile glycerol at −80 °C until use.

### 2.3. Isolation of Bacterial Culture

Bacterial suspensions were obtained from the following dairy matrices: Feta cheese (samples coded as F), Torta del Casar cheese (coded as Q), and various types of kefir (coded as KB and KK). For preincubation, 1 g of each dairy sample was suspended in 24 mL of MRS broth and incubated at 37 °C for 24 h under anaerobic conditions using Mikrobiologie Anaerocult A strips (Sigma-Aldrich (Dublin, Ireland). Subsequently, bacterial isolation was performed by plating the preincubated cultures onto MRS agar using the triple-streak method. After incubation under similar conditions, 60 single colonies exhibiting LAB morphology were selected and inoculated in MRS broth for pH screening. Only isolates that lowered the pH of the culture medium below 4.00 after incubation at 37 °C for 48 h were selected for species-level identification. Microbial identification and characterization were carried out using MALDI-TOF-MS (Matrix-Assisted Laser Desorption/Ionization Time-of-Flight Mass Spectrometry) at the CECT, Valencia, Spain.

### 2.4. Preparation of LAB-Fermented MRS Broth and LAB-Fermented Whey

Sterile MRS broth was prepared according to the manufacturer’s specifications (21 g of MRS per liter of distilled water) and autoclaved at 120 °C for 21 min (Selecta autoclave, Barcelona, Spain). Milk whey, stored at −19 °C, was thawed in a water bath at 50 °C and pasteurized under continuous agitation at 80 °C for 20 min.

A total of 1 mL from a LAB suspension, obtained from a preincubation of LAB-inoculated MRS broth incubated for 24 h at 37 °C, as detailed in Section 2.3, was washed twice with 0.1% (*w*/*v*)peptone/water. Then, 2 mL of the suspension was added into sterile tubes containing either 40 mL of sterile MRS broth or 40 mL of pasteurized milk whey. Control samples were prepared under identical conditions but without the addition of the LAB suspension. All samples were incubated for 72 h at 37 °C under anaerobic conditions. Following incubation, fermentation was terminated by centrifugation of the tubes (3000× *g*, 4 °C, 15 min). The resulting supernatants were collected, frozen (−80 °C, 24 h), lyophilized (FreeZone 2.5 L Benchtop Freeze Dry System, Labconco, Kansas City, MO, USA), and stored at −19 °C, in a freezer (3GIF737F, Balay, Zaragoza, Spain), until further use.

### 2.5. Qualitative Antifungal Activity of Biocontrol Strains

To qualitatively evaluate the antifungal activity of the isolated LAB strains, the overlay assay and the agar diffusion method were performed. In the overlay assay, 20 μL of MRS broth containing each bacterial suspension (prepared 24 h prior) was inoculated onto MRS agar plates and incubated at 37 °C for 72 h to allow for bacterial growth. After incubation, the plates were overlaid with 20 mL of PDA medium previously inoculated with spores from nine selected fungi: *P. camemberti*, *P. expansum*, *P. roqueforti*, *P. digitatum*, *P. brevicopactum*, *P. nordicum*, *P. commune*, *P. solitum* and *P.* A control was performed without the LAB inoculum. After solidification, the plates were incubated 48 h at 25 °C and the appearance of inhibition halos was observed, measured according to the following criteria: (a) without inhibition (−): complete fungal growth covering the bacteria; (b) reduced inhibition (+): 8 mm inhibition halo diameter; (c) medium inhibition (++): formation of marked zones of inhibition around the bacterial inoculum, reaching a 10 mm inhibition halo diameter; strong inhibition (+++): formation of marked zones of inhibition around the bacterial inoculum reaching an inhibition halo diameter of more than 10 mm [25].

For the agar diffusion assay, PDA medium plates were inoculated with the nine *Penicillium* fungal strains used in the overlay assay. Then, using 1 mL micropipette tips, wells were made in the agar and loaded with 50 µL of the fermented culture media, MRS broth or whey, resuspended in sterile water (500 g/L). After incubation (37 °C, 72 h), the appearance of inhibition halos was observed [26].

### 2.6. Quantitative Antifungal Activity of Biocontrol Strains

The quantitative determination of the antifungal activity of the lyophilized whey fermented by LAB isolates was evaluated against nine fungal species of the genus *Penicillium* by the minimum inhibitory concentration–minimum fungicide concentration (MIC-MFC) method described by Luz et al. (2020) [27]. Whey lyophilizates previously fermented with a single LAB strain were resuspended in PDB broth (500 g/L) and serial dilutions (1/2) were prepared into sterile 96-well microplates, obtaining concentrations between 0.49 and 250 g/L with a final volume of 100 µL. Then, 100 µL of PDB liquid medium contaminated with 10^5^ CFU/mL of the *Penicillium* was mixed in each well. A positive control without lyophilized whey was prepared to verify optimal fungal growth, and a negative control (200 µL of uncontaminated PDB culture medium) was prepared to verify the sterility of the culture medium. Each condition was prepared in one microplate column consisting of four replicates (n = 4). The microplates were incubated for 72 h at 25 °C. After the incubation period, the MIC was determined by visual examination of the lowest extract concentration in which a visible growth of the subculture was prevented. Then, 10 μL of each concentration above the MIC was subcultured on PDA plates and incubated for 48 h at 25 °C for the determination of the MFC by the absence of fungal growth after incubation under similar conditions.

### 2.7. Identification of Phenolic Compounds in the Fermented Whey

A QuEChERS extraction was applied for the analysis of phenolic compounds by adding 4 g MgSO_4_, 1 g NaCl, 1% formic acid (*v*/*v*), and 10 mL ethyl acetate to 10 mL of fermented whey. The samples were vortexed for 1 min, incubated on ice for 1 min, and then centrifuged for 10 min at 4 °C and 3000× *g*. The resulting supernatant was mixed with 150 mg C18 and 900 mg MgSO_4_, vortexed for 1 min, and centrifuged again under the same conditions. The final supernatants were evaporated under a nitrogen stream, resuspended in 1 mL of water/acetonitrile (90/10; *v*/*v*), filtered (0.22 µm), and vialized for chromatographic analysis. An Agilent 1200 (Agilent Technologies, Santa Clara, CA, USA) consisting of an autosampler, a binary pump and a vacuum degasser was used for the analysis with a Gemini C_18_ (50 mm × 2 mm, 100 Å, 3 μm size; Phenomenex) column. The mobile phases were water (A) and acetonitrile (B), both acidified with formic acid at 0.1%. The elution gradient was set: 0 min, 5% B; 30 min, 95% B; and 35 min, 5% B; with a flow rate of 0.3 mL min^−1^. A Q-TOF-MS (6540 Agilent ultra-high-definition accurate mass spectrometer), equipped with an Agilent Dual Jet Stream electrospray ionization interface in negative ionization mode, was used for MS analysis, with the following parameters: drying gas flow (N_2_), 8.0 L min^−1^; nebulizer pressure, 30 psig; gas drying temperature, 350 °C; capillary voltage, 3.5 kV; fragmentor voltage, 175 V; and scan range, *m*/*z* 20–380. Collision energies of 10, 20 and 40 eV were used for the MS/MS experiment. Mass hunter Qualitative Analysis Software B.08.00.20 was used for data acquisition and processing [28].

### 2.8. Antifungal Activity of 5% HPMC Coating with LAB-Fermented Whey Lyophilizate

A coating composed of 5% HPMC with LAB-fermented whey lyophilizate was developed in order to be applied in cheese products to inhibit or reduce the growth of cheese fungal spoilage. The antimicrobial activity against *P. commune*, the fungi most typically found in cheese contamination, was evaluated using the LAB isolate that showed higher antifungal activity against this fungal strain, a common contaminant of cheese products.

The control consisted of a HPMC preparation at 5% and PDB broth inoculated with *P. commune* at 2.5 × 10^4^ CFU/mL. The treatments consisted of 5 preparations composed of 5% HPMC, PDB culture broth inoculated with *P. commune* at 2.5 × 10^4^ CFU/mL, and whey lyophilizate fermented by the selected LAB at different concentrations (25 g/L, 37.5 g/L, 50 g/L, 100 g/L and 125 g/L). All were prepared in triplicate.

The preparation was performed following the next steps. First, an HPMC solution at 5% with fermented whey was prepared according to the methodology described by Sousa et al. (2021) with some modifications [29]. The corresponding amount of fermented whey was added to a 39 mL of sterile PDB broth at a temperature of 10 °C. The samples were stirred until complete homogenization and 1 mL of a fungal spore suspension was added. Then, HPMC preparations were obtained by adding 3 g of HPMC to 20 mL of PDB sterile broth at 85 °C until complete dispersion of the mixture (RZR-1 rod stirrer, HEIDOLPH, Schwabach, Germany^®^). Once the HPMC was dispersed, the preparations of 40 mL (containing PDB broth, fermented whey lyophilizate, and the spore suspension) were added, obtaining a final volume of 60 mL. The mixture was stirred until the desired viscosity was achieved. These viscous preparations were incubated at 25 °C under continuous agitation. A count of viable spores was performed after 3, 6, 9 and 12 days of incubation, using the serial dilution method in PDA.

### 2.9. Reduction in Fungal Contamination in Cheese Slices Treated with 5% HPMC Coating LAB-Fermented Whey Lyophilizate

For this assay, fresh cut cheese wedge slices were immersed for 5 s in a coating solution consisting of water, 5% HPMC, and 100 g/L of lyophilized whey extract fermented by LAB. This extract concentration was selected based on previous results. After immersion, excess coating was removed, and the cheese slices were allowed to dry. The coated cheeses were then contaminated by spraying with a *P. commune* suspension to achieve a final concentration of 10^2^ CFU/g of food. Simultaneously, a control sample was prepared using only HPMC without the lyophilized whey extract, followed by the same fungal contamination procedure.

The cheese samples were packed in transparent plastic trays and stored under refrigeration at 4 °C for 30 days. During this period, visual observation of fungal growth on the cheese surface and microbiological analysis of fungal development were conducted on days 10, 20, and 30. For microbiological analysis, the workflow described by Anelli et al. (2024) was followed with slight modifications [30]. Cheese wedges were placed in sterile peptonized water at a 1/10 dilution, homogenized using a stomacher, and 100 µL of the decimal serial dilutions was plated onto PDA medium. The plates were then incubated at 25 °C for 48 h before fungal colony enumeration. The results for the shelf life were expressed as the number of wedges in which fungal contamination was observed. The results from the CFU counting were expressed as mean ± sd.

### 2.10. Reduction in Fungal Contamination in Cheese Treated with 5% HPMC Coating LAB-Fermented Whey Lyophilizate

A final evaluation of the antifungal activity of the developed coating was conducted on whole cheese. Tetilla cheese was selected for this assay. The antifungal properties of the coating were assessed against environmental contamination, without the inoculation of a fungal suspension. The coating was prepared following the procedure described in Section 2.9. After drying, the coated cheeses were stored in sterile trays at 4 °C for 60 days. A total of 4 replicates were used for the assay. Every three days, a visual assessment was performed to monitor mold occurrence. The results were expressed as the percentage of cheeses in which fungal growth was observed.

### 2.11. Statistical Analysis

Statistical analyses for the quantification of phenolic compounds and microbial counts were performed using a one-way ANOVA, followed by Tukey’s HSD post hoc test for multiple comparisons, with a significance level of *p* < 0.05. For the differential analysis of contamination incidence between treatments, data were analyzed using Fisher’s Exact Test at each time point. In trials involving two groups (Control Coated vs. Coat 100 g/L), direct pairwise comparisons were conducted at each time point, and statistical significance was determined at *p* < 0.05. In trials with three groups (Control, Control Coat, and Coat 100 g/L), pairwise comparisons were performed following Fisher’s Exact Test, and *p*-values were adjusted using Bonferroni correction to account for multiple comparisons. Group separation was represented by different letters in the figures; groups sharing the same letter were not significantly different. Asterisks indicate levels of statistical significance in pairwise comparisons: *p* < 0.05 (*), *p* < 0.01 (**), and *p* < 0.001 (***). All statistical analyses were conducted using InfoStat 2019 software (Universidad Nacional de Córdoba, Córdoba, Argentina) and R software 4.3 (GNU Project, Boston, MA, USA).

## 3. Results and Discussion

### 3.1. Isolation, Selection, and Identification of Bacterial Cultures

Of the 60 single colonies initially selected, only 9 isolates remained after the pH and Gram screening. Bacterial identification was performed using MALDI-TOF mass spectrometry (MALDI BioTyper version 3.1 software, MBT 7854 and MBT 7311_RUO Bruker Daltonics databases), which confirmed that all nine isolates belonged to *Lactiplantibacillus plantarum* (*L. plantarum*), with a high probability of species-level identification. The isolates were designated based on their original matrix as F_10_, F_13_, KK_13_, KB_2_, KB_3_, KB_4_, Q_1_, Q_3_, and Q_4_.

### 3.2. Qualitative Antifungal Activity of the LAB

A preliminary screening of the antifungal activity of LAB isolates against nine *Penicillium* species was performed using the dual-culture overlap assay. The isolate with the highest antifungal activity was F_10_, followed by Q_1_, Q_4_, and F_13_. Specifically, F_10_ was effective against all fungal strains, exhibiting marked inhibition halos (++) in all but two strains (*P. roqueforti* and *P. solitum*), where only reduced growth inhibition was observed. Moreover, none of the isolates, except F_10_, were able to inhibit *P. roqueforti* and *P. digitatum*. Isolate Q_1_ was effective against seven fungal strains, with marked inhibition halos observed for *P. expansum*, *P. nordicum*, *P. commune*, and *P. verrucosum*. The isolates Q_4_ and F_13_ exhibited antifungal activity against five *Penicillium* strains, producing inhibition halos classified as medium in three and four of the tested species, respectively. Lower activity was observed for Q_3_, which was effective against four strains, while KB_2_, KB_3_, and KB_4_ inhibited the growth of only three strains. Finally, KK_13_ exhibited antifungal activity solely against *P. expansum* (Table 1).

Numerous studies have evaluated the antifungal efficacy of LAB. Ruggirello et al. (2019) qualitatively assessed the antifungal activity of *L. plantarum* and *Lactobacillus fermentum* strains isolated from cocoa bean fermentation against *Aspergillus flavus* ST2A, *Penicillium citrinum* M6E1TS, *Penicillium griseufulvum* M2BT2 and S2TC, *Aspergillus niger* DsfAn, and *Aspergillus fumigatus* DsfAf, reporting strong inhibition halos [25]. Similarly, Crowley et al. (2013) quantitatively evaluated the antifungal activity of *L. plantarum*, *Weissella confusa*, *Pediococcus pentosaceous* and *Lactococcus lactis* strains against *P. expansum*, *P. digitatum*, *Penicillium notatum*, *P. roqueforti*, *Rhizopus stolonifer*, *Fusarium culmorum*, *Aspergillus fumigatus*, and *Rhodotorula mucilaginosa* [31]. Their study demonstrated that, although all LAB strains exhibited antifungal activity, the two *L. plantarum* strains had the highest antifungal potential, showing inhibition halos of 6–11 mm for *P. digitatum* and *P. roqueforti,* and between 11 and 15 mm for *P. expansum*. The results from this preliminary screening align with previous findings, confirming the antifungal potential of *L. plantarum* strains in inhibiting *Penicillium* spp. growth. It should be noted that the dual-culture overlay method used in this screening is specifically designed to evaluate the antifungal activity of live bacterial cells through direct contact, whereas the antifungal potential of LAB-fermented matrices, attributed to metabolites produced during fermentation, requires alternative approaches such as agar well diffusion assays, which assess the activity of cell-free supernatants [32]. The antifungal effects observed in LAB fermentations are widely described in the literature and are primarily attributed to microbial metabolites. Among the most well-known are lactic acid and phenyllactic acid, although other compounds such as acetic acid, various antimicrobial peptides, and specific substances like nisin and reuterin may also be produced, depending on the bacterial strain, and contribute to the activity [33]. The results from the agar diffusion assays can be observed in Table 2. The fermented MRS lyophilizates that presented the highest antifungal activity were those obtained from isolates F_10_, F_13_, Q_1_, and Q_4_, all of which were active against six fungal strains, showing inhibition halos between 3.4 and 6.6 mm for *P. camemberti* and *P. brevicopactum*, and between 6.7 and 10 mm for *P. nordicum* and *P. verrucosum*. The lyophilized MRS ferments of Q_3_, KB_2_ and KB_3_ showed antifungal activity against five, four and two of the studied strains, respectively, while those fermented by KK_13_ and KB_4_ showed no activity against any fungal strain. On the other hand, the *P. roqueforti*, *P. digitatum and P. solitum* strains were not inhibited by any of the MRS broth lyophilizates fermented by LAB (Table 2a).

The antifungal activity of LAB-fermented whey lyophilizates was clearly enhanced compared with the MRS lyophilizates, as evidenced by the increased inhibition halo diameters against all fungal strains. While the predominant result in the MRS assays was the absence of inhibition, fermented whey lyophilizates exhibited an average inhibition halo diameter of 10 mm. The whey ferments from isolates KB_4_ and Q_1_ demonstrated antifungal activity against eight fungal strains, while all other samples inhibited the growth of all nine tested fungi. The fermented whey lyophilizate obtained from KB_3_ displayed the strongest antifungal activity, with increased inhibition against all fungal strains except for *P. nordicum* and *P. verrucosum*, where the activity remained similar. *P. nordicum* appeared to be the most sensitive fungal strain, with inhibition halo diameters of 11–15 mm observed for LAB-fermented whey lyophilizates from F_10_, F_13_, KK_13_, KB_3_, Q_1_ and Q_3_ (Table 2b).

Since the studied LAB isolates and the whey matrix were both obtained from dairy product sources, the stimulation of the antifungal potential observed when LAB fermentation is performed in whey may be due to specific enzyme synthesis genes and metabolic pathways that are only activated in dairy-based environments [34]. Additionally, whey may contain other compounds that exert a synergistic effect with the antimicrobial metabolites produced by LAB, such as lactoferrin, lysozyme, and the lactoperoxidase system, all of which are well documented in the literature for their antimicrobial activity [35,36]. MRS broth is a selective medium for LAB, providing all necessary nutrients for optimal bacterial growth and fermentation, without exposure to stress or competition from other microbiota. As a result, LAB isolates may not be compelled to produce stress-resistance-related secondary metabolites or antimicrobial substances during the stationary phase. Nevertheless, MRS broth is recognized in the literature as one of the most suitable media for the biosynthesis of LAB-derived bioactive compounds [37]. Therefore, since whey was fermented under identical conditions and exhibited increased antifungal activity, it represents a promising substrate for the production of antifungal agents through LAB fermentation.

Similar results have been previously obtained by other authors. Luz et al. (2020) studied the antifungal potential of MRS broth fermented by *L. plantarum* TR7 and *L. plantarum* TR71 against the same fungal strains studied in the present work, showing antifungal activity against *P. digitatum* CECT 2954 and *P. nordicum* CECT 2320 with similar inhibition halos, as well as no antifungal activity against *P. roqueforti* CECT 2905 [27]. Similarly, the antifungal activity of goat milk whey lyophilizate fermented with *L. plantarum* strains showed inhibition halos equal to or greater than 8 mm for *P. camemberti* 2267 CECT, *P. roqueforti* CECT 2905, *P. verrucosum* VTT-D01847, *P. digitatum* CECT 2954, *P. nordicum* CECT 2320, *P. commune* CECT 20767, *P. solitum* CECT 20818 and *P. pinophilum* CECT 2912, except in the case of the *L. plantarum* CECT 748 strain [38].

### 3.3. Quantitative Antifungal Activity of LAB-Fermented Whey

The MIC and MFC of whey lyophilizates fermented by the studied LAB were determined for all fungal strains. LAB isolates KK_13_, KB_2_, KB_3_ and KB_4_ showed MIC values lower than 62.5 g/L (2.0–62.5 g/L for KK_13_; 3.9–62.5 g/L for KB_2_; 15.6–62.5 g/L for KB_3_), and MIF values lower than 125.0 g/L (3.9–125.0 g/L for KK_13_; 7.8–125.0 g/L for KB_2_ and KB_4_; 31.3–125.0 g/L for KB_3_) except for *P. camemberti* (MIC: 125.0 g/L; MFC: 250.0 g/L). Q_3_ and Q_4_ showed MIC values between 31.3 and 125 g/L and MFC values between 62.5 and 250.0 g/L (except *P. digitatum*). Finally, the MIC values ranged from 62.5 to 125.0 g/L for Q_1_, F_10_ and F_13_ (except *P. digitatum*; MIC = 250.0 g/L), with MFC ranging from 125.0 to 250.0 g/L (Table 3).

**Table 3:** MIC and MFC values (g/L) obtained from the lyophilizates of whey fermented by the selected LAB against nine fungal strains of the genus *Penicillium*. The abbreviations F_10_, F_13_, KK_13_, KB_2_, KB_3_, KB_4_, Q_1_, Q_3_, and Q_4_ correspond to the different *Lactiplantibacillus plantarum* isolates evaluated in this study.

Fungal Strain	LAB Strain																	
	F_10_		F_13_		KK_13_		KB_2_		KB_3_		KB_4_		Q_1_		Q_3_		Q_4_	
	MIC	MFC	MIC	MFC	MIC	MFC	MIC	MFC	MIC	MFC	MIC	MFC	MIC	MFC	MIC	MFC	MIC	MFC
***P. camemberti***	125.0	>250.0	62.5	125.0	125.0	250.0	125.0	250.0	125.0	250.0	125.0	250.0	125.0	250.0	125.0	250.0	125.0	250.0
***P. expansum***	62.5	125.0	62.5	125.0	31.3	62.5	62.5	125.0	62.5	125.0	31.3	62.5	125.0	250.0	62.5	125.0	62.5	125.0
***P. roqueforti***	125.0	250.0	62.5	125.0	2.0	3.9	3.9	7.8	15.6	31.3	3.9	7.8	125.0	250.0	62.5	125.0	31.3	62.5
***P. digitatum***	250.0	>250.0	125.0	250.0	62.5	125.0	62.5	125.0	62.5	125.0	62.5	125.0	62.5	125.0	250.0	>250.0	125.0	250.0
***P. brevicopactum***	125.0	250.0	62.5	125.0	31.3	62.5	31.3	62.5	31.3	62.5	31.3	62.5	62.5	125.0	62.5	125.0	62.5	125.0
***P. nordicum***	125.0	250.0	62.5	125.0	31.3	62.5	31.3	62.5	15.6	31.3	31.3	62.5	125.0	250.0	62.5	125.0	31.3	62.5
***P. commune***	125.0	250.0	62.5	125.0	15.6	31.3	31.3	62.5	15.6	31.3	31.3	62.5	125.0	250.0	62.5	125.0	125.0	250.0
***P. solitum***	125.0	250.0	62.5	125.0	62.5	125.0	62.5	125.0	31.3	62.5	125.0	250.0	125.0	250.0	31.3	62.5	62.5	125.0
***P. verrucosum***	125.0	250.0	62.5	125.0	62.5	125.0	62.5	125.0	62.5	125.0	62.5	125.0	125.0	250.0	62.5	125.0	31.3	62.5
**Mean**	**MIC**	**MFC**	**MIC**	**MFC**	**MIC**	**MFC**	**MIC**	**MFC**	**MIC**	**MFC**	**MIC**	**MFC**	**MIC**	**MFC**	**MIC**	**MFC**	**MIC**	**MFC**
	131.9	236.1	69.4	138.9	47.1	94.2	52.5	105.0	46.9	93.7	56.0	112.0	111.1	222.2	86.8	145.8	78.1	145.8

The MIC and MFC results showed the highest antifungal activity for whey lyophilizates fermented by KK_13_, KB_2_ KB_3_ and KB_4_, confirming the hypothesis that fermented whey is capable of enhancing the fungicidal action of certain *L. plantarum* strains. Of all these LAB, the whey lyophilizate fermented by KB_3_ was able to inhibit the growth of *P. commune* CETC 20767 with an MIC of 15.63 g/L and an MFC of 31.25 g/L (Table 3). A tendency also observed in the agar diffusion assay. Therefore, it was selected to carry out further antifungal activity studies of coating intended to be applied in cheese products.

There are limited studies in the literature regarding the antifungal activity of whey fermented by LAB. Izzo et al. (2020) obtained similar MIC and MFC values of goat whey lyophilizates fermented with *L. plantarum* strains against *P. nordicum* CECT 2320 and *P. commune* CECT 20767 [38]. Specifically, *L. plantarum* CECT 220 and *L. plantarum* CECT 748 showed an MIC of 62.5 g/L and an MFC of 125 g/L, while *L. plantarum* CECT 221 and *L. plantarum* CECT 223 obtained an MIC of 31.2 g/L and an MFC of 250 and 125 g/L, respectively.

### 3.4. Identification of Phenolic Compounds in the Fermented Whey

The presence of phenolic compounds was analyzed in the fermented whey samples, as some of the organic compounds potentially responsible for the observed antifungal activity (Table 4). Among the targeted antifungal compounds, six phenolic compounds were detected in significant quantities in the whey extracts fermented by different LAB strains. The most abundant compound was DL-3-phenyllactic acid, present in all LAB ferments at concentrations of up to 3.92 mg/L, followed by benzoic acid (up to 2.08 mg/L), 3,4-dihydroxyhydrocinnamic acid (up to 2.22 mg/L), and vanillic acid (up to 0.09 mg/L), which were found in the majority of the fermented extracts. Other compounds, such as 1,2-dihydroxybenzene and 3-(4-hydroxy-3-methoxyphenyl)propionic acid, were detected in lower concentrations and only in whey fermented by the Q_3_ isolate. In agreement with the antifungal activity results, whey fermented by the KB_3_ isolate exhibited significantly higher concentrations of the most abundant phenolic compounds, the DL-3-phenyllactic acid, 3-4-dihydroxyhydrocinnamic and benzoic acid, all of which have been identified in the literature as antimicrobial agents [39,40].

When studying the antifungal properties of natural extracts fermented by *Lactobacillus* spp. strains, similar phenolic compounds were identified, highlighting compounds such as DL-3-phenyllactic acid and benzoic acid, as in the present work [41,42]. These phenolic compounds have been previously reported as antifungal agents produced by LAB, and it was found that greater phenolic acid production by the different LAB strains correlated with a higher antifungal activity of fermented matrices [26]. Previous studies have reported MIC values for these compounds in pure form at much higher concentrations than those found in the present study, such as an MIC of 0.5 g/L for pure 3-phenyllactic [39]. However, a survey in the literature suggests that the antifungal activity observed in LAB fermentation is the result of a synergistic effect from the diverse metabolite pool produced by these bacteria, such as the one presented in this work by the KB_3_ isolate [33].

### 3.5. Antifungal Activity of 5% HPMC Coating with LAB-Fermented Whey

**Figure 1:**
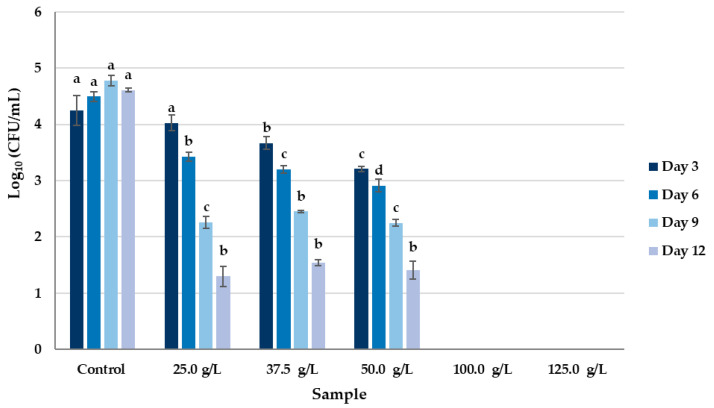
Reduction in fungal growth of *P. commune* inoculated in coatings composed of PDA culture broth, 5% HPMC, and varying doses (25 g/L, 37.5 g/L, 50 g/L, 100 g/L, and 125 g/L) of KB_3_-fermented whey lyophilizate. Different letters indicate statistically significant differences among sample means on the same day (*p* < 0.05).

The 5% HPMC preparations with 100 g/L and 125 g/L of KB_3_-fermented whey lyophilizate completely inhibited the growth of *P. commune* from day 3. After carrying out counting on day 12, no statistically significant differences were observed between the coatings with 25 g/L, 37.5 g/L, 50 g/L of whey fermented lyophilizate. However, there was a significant (*p* < 0.05) fungal growth reduction compared with the control. Specifically, on day 12, the coatings with 25 g/L, 37.5 g/L and 50 g/L of KB_3_ fermented whey lyophilizate showed a significative reduction of 3.01, 3.07 and 3.20 Log_10_ CFU/L compared with the control, respectively. Moreover, a significant reduction in the CFU/mL from the concentrations above 37.5 g/L was observed after the 6th day in comparison to the control (Figure 1).

Many studies indicate how LAB produce antimicrobial substances during fermentation [43]. Therefore, the antifungal capacity of the KB_3_-fermented whey lyophilized coating may be attributed to the presence of antimicrobial substances in whey, such as organic acids and peptides produced during KB_3_ fermentation, which, together with the presence of bioactive compounds naturally present in whey, may contribute to the coating antifungal properties. Few studies in the literature have explored the use of LAB and whey in edible coatings for biopreservation purposes. For example, Guimarães et al. (2020) shows how the addition of *L. buchneri* UTAD104 in coatings composed by whey protein concentrate, glycerol, guar gum and Tween 20 managed to inhibit the fungal growth of 1 × 10^6^ spores/mL of *Penicillium nordicum* in 30 days [44]. Other studies also investigated the potential use from other edible coatings, such as that of Azhdari & Moradi (2022), that reported the development of a carboxymethyl cellulose coating with natamicina that inhibited the growth of *A. flavus*, *A. fumigatus*, *A. niger*, *P. citrinum*, and *C. albicans* in in vitro assays [45]. The authors demonstrated the effectiveness of the coating in agar diffusion assays, where natamycin at a concentration of 0.5% produced inhibition halos of approximately 3 cm. The use of specific antifungal compounds in edible coatings is well documented in the literature. However, the isolation of these compounds often involves organic solvents and leads to increased production costs [46]. In contrast, the coating studied in the present work offers an eco-friendlier and more cost-effective alternative, supporting the feasibility of implementing this technology in the food industry.

### 3.6. Reduction in Fungal Contamination in Cheeses Treated with 5% HPMC Coating LAB-Fermented Whey

**Figure 2:**
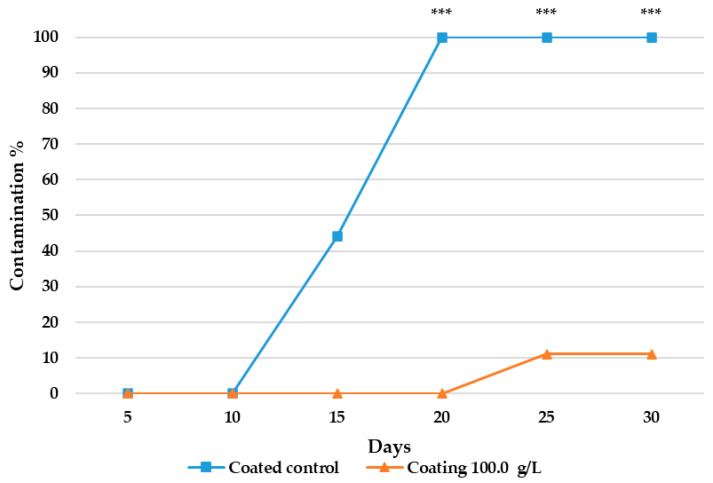
The results of the shelf-life assays in cheese wedges contaminated with *P. commune*. Treatments include control cheeses (coated control) and cheeses coated with a gel composed of 5% HPMC in water and 100 g/L of KB_3_-fermented whey lyophilizate (coating 100 g/L). Statistically significant differences in pairwise comparisons are indicated by asterisks: *p* < 0.001 (***).

**Figure 3:**
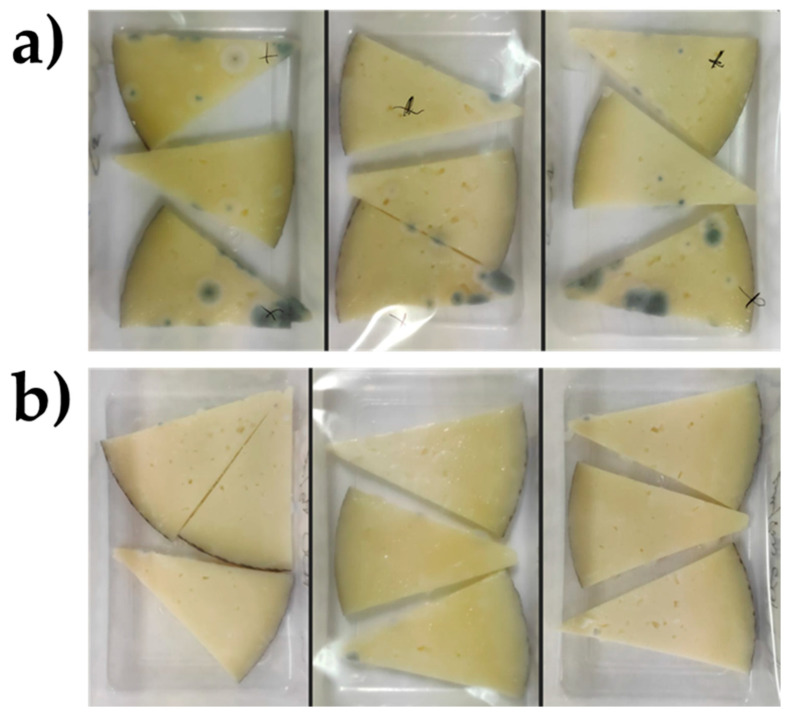
Appearance of cheese wedges contaminated with *P. commune* after 20 days of storage at refrigerated temperature (4 °C): (**a**) control cheese and (**b**) cheese treated with 5% HPMC coating containing 100 g/L of KB_3_-fermented whey lyophilizate.

Visual control of cheese slices contaminated with *P. commune* and kept at a refrigerated temperature (4 °C) revealed that no fungal growth was observed in the untreated control cheeses until day 10, while in the case of cheeses treated with KB_3_ fermented whey extract at 100 g/L, visible fungal growth did not begin until day 22 (Figure 2). Fisher’s Exact Test confirmed that the observed differences became statistically significant after day 20 of the assay. An example the visual evaluation of the fungal growth is shown in Figure 3.

**Figure 4:**
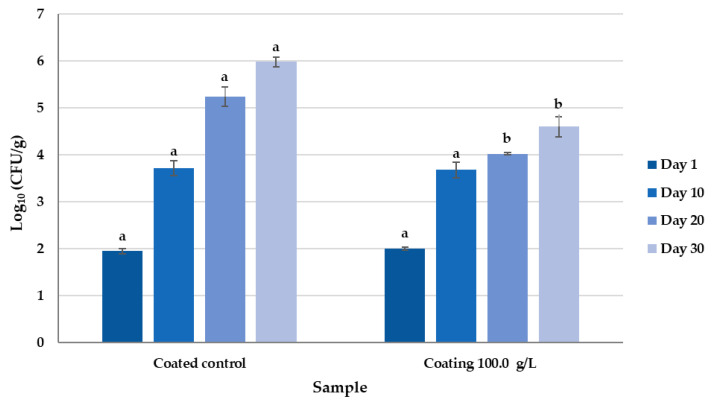
Fungal growth of *P. commune* inoculated on control cheeses (coated control) and cheeses coated with a gel composed of 5% HPMC in water and 100 g/L of KB_3_-fermented whey lyophilizate (coating 100 g/L). Different letters indicate statistically significant differences among sample means on the same day (*p* < 0.05).

As shown in Figure 4, the microbiological count of the cheeses at day 10 showed no significant differences between the treated and control samples. However, by day 20, a reduction in the fungal count of 1.22 Log_10_ CFU/g in the treated cheeses (4.08 Log_10_ CFU/g) compared with the controls (5.3 Log_10_ CFU/g) was observed. This reduction was maintained until the end of the trial (day 30), where the treated cheeses exhibited a fungal load of 4.60 Log_10_ CFU/g, representing a 1.38 Log_10_ CFU/g decrease compared with the controls (5.9 Log_10_ CFU/g).

**Figure 5:**
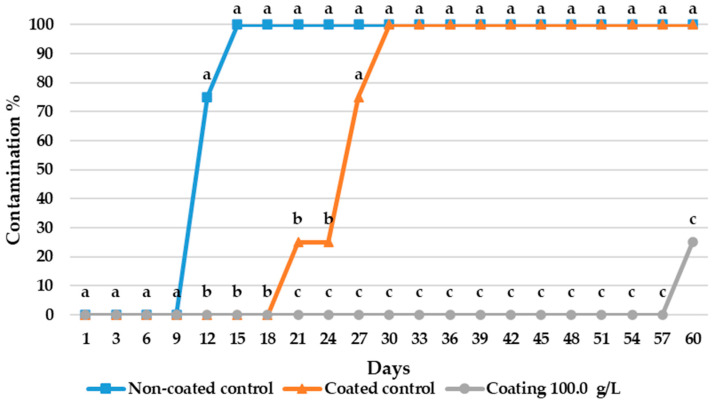
The results of the shelf-life analysis assays of whole cheeses with natural contamination. Treatments include cheese without coating (non-coated control), control cheeses coated with 5% HPMC in water (coated control), and cheeses coated with a gel composed of 5% HPMC in water and 100 g/L of KB_3_-fermented whey lyophilizate (coating 100 g/L). Significant differences among the samples are indicated by different letters; comparisons were performed for each day (*p* < 0.05). When identical values showed no statistical difference, the letter was used only once to avoid redundancy.

**Figure 6:**
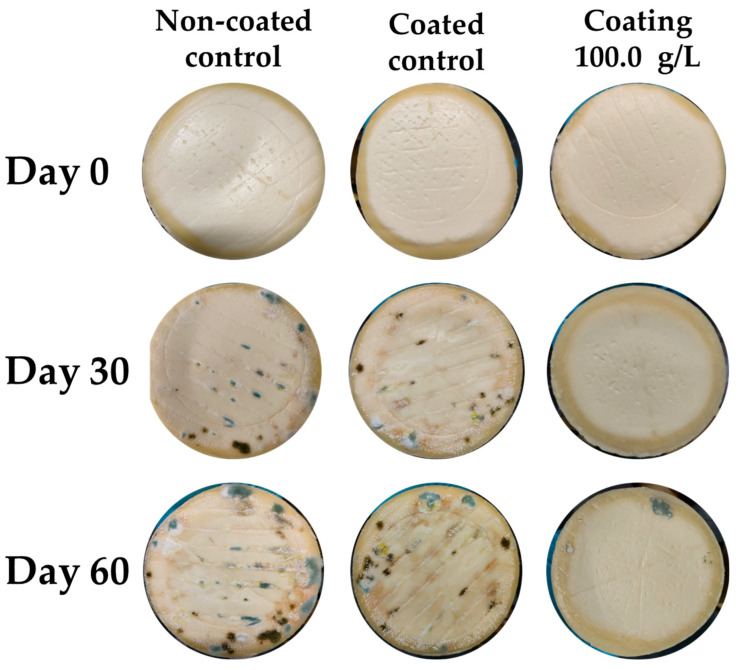
Whole cheeses after 0, 30, and 60 days of incubation in the natural contamination assays, stored at a refrigerated temperature (4 °C). The treatments applied in this assay were as follows: non-coated control, coated control, and coating with 100.0 g/L of KB_3_-fermented whey lyophilizate.

A visual evaluation of the coating’s effects on whole cheese is presented in Figure 5. The results indicate that fungal spots first appeared after 12 days of incubation in the control samples without any coating. The addition of the control coating delayed fungal growth until day 27 of the assay. Finally, the treatment incorporating the coating with KB_3_-fermented whey extract successfully extended the onset of fungal growth until day 60, increasing the product’s shelf life by a total of 45 days compared with the untreated control samples, and by 33 days compared with the coated control samples. Statistical tests revealed that after 21 days of incubation, the samples treated with the KB_3_ coating were significantly different from both control groups. Therefore, it can be concluded that the treatment was effective in delaying fungal growth. An example of how the evaluation was performed can be observed in Figure 6.

Previous studies have demonstrated the use of LAB as biopreservatives in cheese matrices. Other studies have investigated the biopreservative application of whey fermented by a *L. plantarum* strain as a coating applied to plastic film separators for cheese slices [28]. This work reported that the use of this film delayed the visual occurrence of *P. commune* a total of 15 days compared with a control. Vasiliauskaite et al. (2023) used an approach similar to the one followed in this article: an acid whey protein concentrate fermented by *Lacticaseibacillus paracasei* sp. with pectin as a gelling agent that reduced the presence of fungi after a 14-day incubation in 1.00 Log_10_ CFU/g of food [47]. These findings highlight the promising role of fermented whey as a natural and effective strategy for extending the shelf life of cheese products by inhibiting fungal growth. The ability of LAB-fermented whey to be incorporated into various preservation systems, such as coating films and gelled matrices, demonstrates its versatility and potential applicability in the dairy industry. By leveraging the antimicrobial properties of LAB strains, these biopreservation approaches provide a sustainable alternative to conventional antifungal treatments, reducing the reliance on chemical preservatives while maintaining product quality and safety. Furthermore, converting whey into a functional biopreservative contributes to waste valorization, offering an environmentally friendly solution to both food preservation and the reduction in dairy industry waste.

## 4. Conclusions

In this study, several *Lactiplantibacillus plantarum* strains demonstrated antifungal activity in vitro against *Penicillium* species commonly responsible for cheese spoilage. Among the tested media, whey-based fermentations showed enhanced antifungal activity compared to MRS fermentations, confirming whey as a valuable natural substrate for bio-preservation. This highlights the potential of whey not only as a fermentation medium but also as a bioactive ingredient, transforming an environmentally burdensome dairy by-product into a functional component with significant antifungal properties. Phenolic compound analysis supported these findings, revealing key antifungal metabolites such as phenyllactic acid, particularly abundant in whey fermented by strain KB_3_. The application of a coating composed of 5% HPMC and KB_3_-fermented whey lyophilizate effectively reduced fungal growth on cheese and extended shelf life by 12 days under controlled contamination. Moreover, trials conducted on whole cheeses with natural contamination demonstrated a delay in visible fungal growth until day 60, extending shelf life by 45 days compared to untreated samples and by 33 days relative to coatings without the active ferment.

Overall, coatings based on LAB-fermented whey and HPMC offer a promising, sustainable alternative to synthetic preservatives for extending cheese shelf life. This strategy not only enhances food quality and safety but also supports circular economy principles by valorizing dairy by-products and contributing to the reduction in plastic packaging in the food industry.

## Figures and Tables

**Table 1:** Qualitative overlay assay of the antifungal activity of LAB against fungi of the genus *Penicillium*. Fungal growth inhibition was assessed after 48 h. The results are expressed as follows: no inhibition (−), reduced inhibition (+), where bacterial growth was not overlapped by the mold but was in close proximity; and medium inhibition (++). The non-bacterial control is indicated as C. The abbreviations F_10_, F_13_, KK_13_, KB_2_, KB_3_, KB_4_, Q_1_, Q_3_, and Q_4_ correspond to the different *Lactiplantibacillus plantarum* isolates evaluated in this study.

Fungal Strain	Samples									
	C	F_10_	F_13_	KK_13_	KB_2_	KB_3_	KB_4_	Q_1_	Q_3_	Q_4_
*P. camemberti*	−	++	+	−	−	++	+	+	−	+
*P. expansum*	−	++	+	++	−	−	−	++	−	++
*P. roqueforti*	−	+	−	−	−	−	−	−	−	−
*P. digitatum*	−	++	−	−	−	−	−	−	−	−
*P. brevicopactum*	−	++	+	−	+	+	+	+	+	−
*P. nordicum*	−	++	++	−	+	++	++	++	+	++
*P. commune*	−	++	++	−	++	−	−	++	+	−
*P. solitum*	−	+	−	−	−	−	−	+	++	++
*P. verrucosum*	−	++	−	−	−	−	−	++	−	+

**Table 2:** Antifungal agar diffusion activity assay of (**a**) MRS broth and (**b**) whey fermented with LAB against various fungal strains of the genus *Penicillium*, assessed by the agar diffusion method. Inhibition zones are categorized by diameter, as follows: 0 mm (−), 1–3.3 mm (+), 3.4–6.6 mm (++), and 6.7–10 mm (+++). The non-bacterial control is indicated as C. The abbreviations F_10_, F_13_, KK_13_, KB_2_, KB_3_, KB_4_, Q_1_, Q_3_, and Q_4_ correspond to the different *Lactiplantibacillus plantarum* isolates evaluated in this study.

**(a) MRS**										
**Fungal Strain**	**LAB Strain**									
	**C**	**F_10_**	**F_13_**	**KK_13_**	**KB_2_**	**KB_3_**	**KB_4_**	**Q_1_**	**Q_3_**	**Q_4_**
*P. camemberti*	−	++	++	−	−	−	−	++	+	++
*P. expansum*	−	+	+	−	−	−	−	+	−	+
*P. roqueforti*	−	−	−	−	−	−	−	−	−	−
*P. digitatum*	−	−	−	−	−	−	−	−	−	−
*P. brevicopactum*	−	++	++	−	+	+	−	++	+	++
*P. nordicum*	−	+++	+++	−	+	−	−	+++	++	+++
*P. commune*	−	+	+	−	+	−	−	+	+	+
*P. solitum*	−	−	−	−	−	−	−	−	−	−
*P. verrucosum*	−	+++	+++	−	+	++	−	+++	++	+++
**(b) Whey**										
**Fungal Strain**	**LAB Strain**									
	**C**	**F_10_**	**F_13_**	**KK_13_**	**KB_2_**	**KB_3_**	**KB_4_**	**Q_1_**	**Q_3_**	**Q_4_**
*P. camemberti*	−	++	++	++	++	+++	++	−	+++	++
*P. expansum*	−	++	++	++	++	++	++	++	++	++
*P. roqueforti*	−	+	++	++	++	++	++	++	++	++
*P. digitatum*	−	+	++	+	++	++	−	++	+	+
*P. brevicopactum*	−	+	++	+	+	++	+	+	+	+
*P. nordicum*	−	+++	+++	+++	++	+++	++	+++	+++	++
*P. commune*	−	++	++	++	+	++	++	++	++	++
*P. solitum*	−	++	++	++	+	++	++	++	++	++
*P. verrucosum*	−	+++	++	++	++	+++	++	++	++	++

**Table 4:** Identification and quantification of phenolic compounds (mg/L) produced by LAB in fermented whey. The non-bacterial control is indicated as C. The abbreviations F_10_, F_13_, KK_13_, KB_2_, KB_3_, KB_4_, Q_1_, Q_3_, and Q_4_ correspond to the different *Lactiplantibacillus plantarum* isolates evaluated in this study. tHE Identified phenolic compounds are grouped as follows: (**a**) DL-3-phenyllactic acid, 3,4-dihydroxyhydrocinnamic acid, and benzoic acid; (**b**) vanillic acid, 1,2-dihydroxybenzene, and 3-(4-hydroxy-3-methoxyphenyl)propionic acid. Significant differences among samples are indicated by different letters (*p* < 0.05).

**(a)**			
**Sample**	**DL-3-Phenyllactic Acid**	**3-4-Dihydroxyhydrocinnamic**	**Benzoic Acid**
**C**	nd	nd	nd
**F_10_**	3.26 ± 0.44 ^a^	1.26 ± 0.15 ^b^	1.22 ± 0.15 ^b^
**F_13_**	2.70 ± 0.20 ^b^	nd	0.82 ± 0.15 ^c^
**KK_13_**	0.18 ± 0.07 ^d^	0.15 ± 0.03 ^c^	nd
**KB_2_**	0.24 ± 0.08 ^d^	nd	0.17 ± 0.08 ^d^
**KB_3_**	3.92 ± 0.07 ^a^	2.22 ± 0.02 ^a^	2.08 ± 0.15 ^a^
**KB_4_**	0.79 ± 0.08 ^c^	0.19 ± 0.09 ^c^	0.11 ± 0.04 ^d^
**Q_1_**	3.41 ± 0.21 ^a^	2.12 ± 0.11 ^a^	1.42 ± 0.51 ^b^
**Q_3_**	0.28 ± 0.02 ^d^	0.30 ± 0.03 ^c^	0.09 ± 0.01 ^d^
**Q_4_**	3.02 ± 0.68 ^a^	0.21 ± 0.09 ^c^	0.10 ± 0.02 ^d^
**(b)**			
**Sample**	**Vanillic Acid**	**1-2-Dihydroxybenzene**	**3-(4-hydroxy-3-methoxyphenyl) Propionic**
**C**	nd	nd	nd
**F_10_**	nd	nd	nd
**F_13_**	nd	nd	nd
**KK_13_**	0.09 ± 0.03 ^a^	nd	nd
**KB_2_**	0.09 ± 0.03 ^a^	nd	nd
**KB_3_**	0.04 ± 0.03 ^a^	nd	nd
**KB_4_**	0.06 ± 0.05 ^a^	nd	nd
**Q_1_**	0.07 ± 0.02 ^a^	nd	nd
**Q_3_**	0.08 ± 0.02 ^a^	0.06 ± 0.01 ^a^	0.04 ± 0.01 ^a^
**Q_4_**	0.09 ± 0.03 ^a^	nd	nd

## Data Availability

The original contributions presented in this study are included in this article. Further inquiries can be directed to the corresponding author.

## Abbreviations

The following abbreviations are used in this manuscript:

CECT	Spanish Type Culture Collection
F_10_	*Lactiplantibacillus plantarum* F_10_
F_13_	*Lactiplantibacillus plantarum* F_13_
HPMC	Hydroxypropylmethylcellulose
KB_2_	*Lactiplantibacillus plantarum* KB_2_
KB_3_	*Lactiplantibacillus plantarum* KB_3_
KB_4_	*Lactiplantibacillus plantarum* KB_4_
KK_13_	*Lactiplantibacillus plantarum* KK_13_
LAB	Lactic acid bacteria
MIC	Minimum inhibitory concentration
MFC	Minimum fungicide concentration
MRS	Man–Rogosa–Sharpe
PDA	Potato dextrose agar
PDB	Potato dextrose broth
Q_1_	*Lactiplantibacillus plantarum* Q_1_
Q_3_	*Lactiplantibacillus plantarum* Q_3_
Q_4_	*Lactiplantibacillus plantarum* Q_4_
